# Bilateral simultaneous cochlear implantation is a safe method of hearing rehabilitation in adults

**DOI:** 10.1007/s00405-023-07977-z

**Published:** 2023-05-16

**Authors:** Maximilian Gröger, Andreas Loth, Silke Helbig, Timo Stöver, Martin Leinung

**Affiliations:** University Hospital Frankfurt, Dept. of Otorhinolaryngology, Frankfurt am Main, Germany

**Keywords:** Bilateral cochlear implant, Adults, Simultaneous, Sequential, Complication, Total operating room time

## Abstract

**Purpose:**

Bilateral cochlear implantation is an effective treatment for patients with bilateral profound hearing loss. In contrast to children, adults mostly choose a sequential surgery. This study addresses whether simultaneous bilateral CI is associated with higher rates of complications compared to sequential implantation.

**Methods:**

169 bilateral CI surgeries were analyzed retrospectively. 34 of the patients were implanted simultaneously (group 1), whereas 135 patients were implanted sequentially (group 2). The duration of surgery, the incidence of minor and major complications and the duration of hospitalization of both groups were compared.

**Results:**

In group 1, the total operating room time was significantly shorter. The incidences of minor and major surgical complications showed no statistically significant differences. A fatal non-surgical complication in group 1 was particularly extensively reappraised without evidence of a causal relationship to the chosen mode of care. The duration of hospitalization was 0.7 days longer than in unilateral implantation but 2.8 days shorter than the combined two hospital stays in group 2.

**Conclusion:**

In the synopsis of all considered complications and complication-relevant factors, equivalence of simultaneous and sequential cochlear implantation in adults in terms of safety was found. However, potential side effects related to longer surgical time in simultaneous surgery must be considered individually. Careful patient selection with special consideration to existing comorbidities and preoperative anesthesiologic evaluation is essential.

## Introduction

Cochlear implantation (CI) is the internationally established ‘gold standard’ for the therapy of profound hearing impairment up to deafness [1, 2]. After the introduction of this method into clinical use, the reliability of the implants and the safety of the surgical technique were successively improved over the following decades [3, 4]. This led to steadily decreasing complication rates since the 1990s and made the surgery, initially performed exclusively unilaterally, a largely safe procedure [5, 6].

Functional usability of both ears significantly improves speech understanding, both in quiet and in noise [7]. In bilateral CI use, the separation of noise and signals is optimized due to psychoacoustic phenomena such as head shadow effect [8], squelch effect [9] and binaural summation [10, 11]. Thus, bilateral CI therapy is a widely common procedure in many countries.

Both children and adults benefit from early and binaural CI fitting when indicated: advantages of bilateral CIs have been demonstrated for speech understanding in noise, subjective impairment due to tinnitus, and perceived "auditory stress" [12, 13]. The advantages of bilateral CI therapy remain stable over time [14]. This could have a positive effect on cognitive performance in older age [15], whereas an insufficiently provided age-related hearing loss and consecutive social isolation can promote dementia [16, 17].

From a purely technical perspective, bilateral cochlear implantation can be performed either simultaneously or sequentially. Initially, there were objections about the simultaneous procedure—in particular—the postoperative balance function, the risk of extensive anesthesia time, and cost-effectiveness [18, 19]. A first larger case series of simultaneous bilateral implantations was initially examined in children [20], where no adverse events were reported. For infants the safety of this procedure was confirmed also by others [21, 22].

In contrast to children, most adult patients with bilateral hearing loss opt for a sequential surgical approach mostly starting with the worse hearing ear. After successful hearing rehabilitation of the first ear, CI therapy of the second ear often follows.

A frequently mentioned argument against a simultaneous bilateral procedure is a possibly increased risk of perioperative complications due to the prolonged duration of anesthesia or because of prolonged hospitalization. For children, it has already been demonstrated that simultaneous care is a low-risk and resource-saving procedure [23]. However, at present data on adult patients (> 18 years) is rare. Thus, the aim of this study was to evaluate the safety of simultaneous versus sequential bilateral CI surgery in adults. For this, the incidence of minor and major complications, the duration of surgery and the length of hospital stay were examined, including a long term follow up.

## Materials and methods

The presented work is a retrospective analysis of patient data performed with approval of the local ethical committee (reference number 580/20). All patients who received simultaneous or sequential bilateral cochlear implantation between 2008 and 2016 in our hospital were included in the study. The postoperative course was evaluated until the end of 2021. Thus, each patient was followed up for at least 5 years. Additional inclusion criteria were the presence of bilateral postlingual deafness or profound hearing loss and age > 18 years at the time of the first surgery. Patients with previous ear or CI surgery were excluded. In total, 169 bilaterally implanted patients were included. 34/169 (20.1%) individuals received simultaneous bilateral implantation (group 1) and 135/169 (79.9%) received sequential CI surgery (group 2). Patient data were extracted from a proprietary hearing implant database and supplemented with the relevant clinical data (medical and nursing documentation, laboratory blood results, surgical and anesthesiologic protocols) of the hospital information system ORBIS® (Dedalus Healthcare GmbH, Bonn).

Three different parameters were determined to compare sequential and simultaneous CI surgery: (1) the surgery time, (2) the occurrence of intraoperative or postoperative complications, and (3) the duration of hospitalization.

### Duration of surgery

The total operating room time (TORT), the pure “surgical time” and the time required for anesthesiologic preparation and post-processing (“setup time” were extracted from the hospital information system. The TORT is defined by the room time (in minutes) from the start of the anesthesiologic measures until the patient is transferred to the recovery room. The surgical time is equivalent to the “incision-suture time”. The time interval from patient arrival in the operating room to skin incision (set-up time) includes anesthesiologic measures (e.g. intubation), patient positioning, and team timeout procedure.

### Intra- and postoperative complications

Adverse events were primarily differentiated according to surgical and non-surgical complications. A modified version of the definition of CI-related complications by Farinetti et al. [24] was used in this study. It was differentiated between minor and major surgical complications: minor complications are transient and can be managed conservatively, whereas major complications require a new surgical procedure or impose permanent restrictions on the patient (see Table 1).

**Table 1:** List of possible non-surgical and surgical major and minor complications of cochlear implantation

Non-surgical complications	Hypoxemia Hypercapnia Aspiration Intubation error Hypertensive crisis	Deep vein thrombosis Pulmonary embolism Catecholamine liability Duty to resuscitate Exitus letalis
Surgical complications		
Minor	Transient taste disorder Transient vertigo Transient tinnitus Facial costimulation	Significant pain^a^ Low-grade wound dehiscence^b^ Edema/hematoma over implant bed
Major	Facial nerve palsy Meningitis CSF Cholesteatoma Tympanic membrane perforation Implant failure	Extracochlear insertion Electrode migration Severe wound dehiscence^c^ Discontinuation of surgery due to relevant Hb decrease Opening of the auditory canal

^a^Analgesia longer than one week and/or need for pain consultation

^b^Minor = conservatively manageable

^c^Severe = operative revision required

Minor complications following bilateral CI surgery often were not clearly localized to one surgical site (e.g., postoperative vertigo with simultaneous implantation). Therefore, the incidence of minor complications was related to the number of patients or surgical procedures. Major complications, on the other hand, could usually be clearly localized (implant failure, electrode migration, magnet dislocation) and were thus evaluated in relation to the number of implants.

Non-surgical complications were hypoxemia, hypercapnia, aspiration, intubation failure, nosocomial pneumonia, hypertensive crisis, deep vein thrombosis, pulmonary embolism, catecholamine requirement, resuscitation requirement and death.

Data collection was performed until the end of 2021 resulting in a follow-up period of all included patients of at least 5 years and a maximum of 13 years. This design was chosen to detect long time complications.

### Duration of hospitalization

The duration of hospitalization for CI surgery was defined as the number of postoperative days, excluding the day of surgery. Discharge on the third postoperative day represented the standard procedure in routine clinical practice. Patients with a later discharge date were regarded as "overstayers" and evaluated separately.

All data were merged and statistically analyzed in Microsoft^®^ Excel^®^ 2016 (Microsoft Corporation, Redmond, USA). Surgical time and duration of hospitalization were tested for normal distribution using the Kolmogorov–Smirnov test, expressed as means ± standard deviation, and tested for differences in means using the two-sample *t* test. Complications were counted in both groups, and contingency tables were tested for independence using the chi-square test. Due to small numbers of major complications, Fisher's exact test was used, which is most appropriate for small sample sizes. The criterion for statistical significance (marked with * in figures) was a *p* value of ≤ 0.05 (** = high significance with *p* ≤ 0.01).

## Results

The average patient age in group 1 was 50.8 ± 12.7 years (mean ± standard deviation) with an age range of 20.1 to 74.1 years. Patients in group 2 were 52.3 ± 15.7 years old at the time of the first operation (age range 19.6–84.1 years). The gender ratio within the groups was also comparable with a slight predominance of women over men (men: 22/34 = 65% in group 1, 77/135 = 57% in group 2. Even regarding the distribution of the different implant manufacturers, both groups showed strong similarities—despite different group sizes: in both groups, half of the patients were implanted with a device from the manufacturer Cochlear Ltd (Sydney, Australia; 18/34 = 53% group 1, 62/135 = 46% group 2), closely followed by MED-EL (Innsbruck, Austria; 15/34 = 44% group 1, 65/135 = 48% group 2). Systems from the manufacturer Advanced Bionics (Valencia, USA) were used only sporadically (1/34 = 3% group 1, 8/135 = 6% group 2).

### Duration of surgery

The duration of the setup time was 66 ± 16 min in group 1, which was significantly (*p* < 0.05) longer than the setup time of the first surgery of the sequentially supplied group 2 (60 ± 17 min). The difference to the surgery of the second ear in group 2 was highly significant (59 ± 15 min; *p* < 0.01). The total setup time of both ears in group 2 (119 ± 26 min) was highly significant above the setup time of group 1 (*p* < 0.01).

**Fig. 1:**
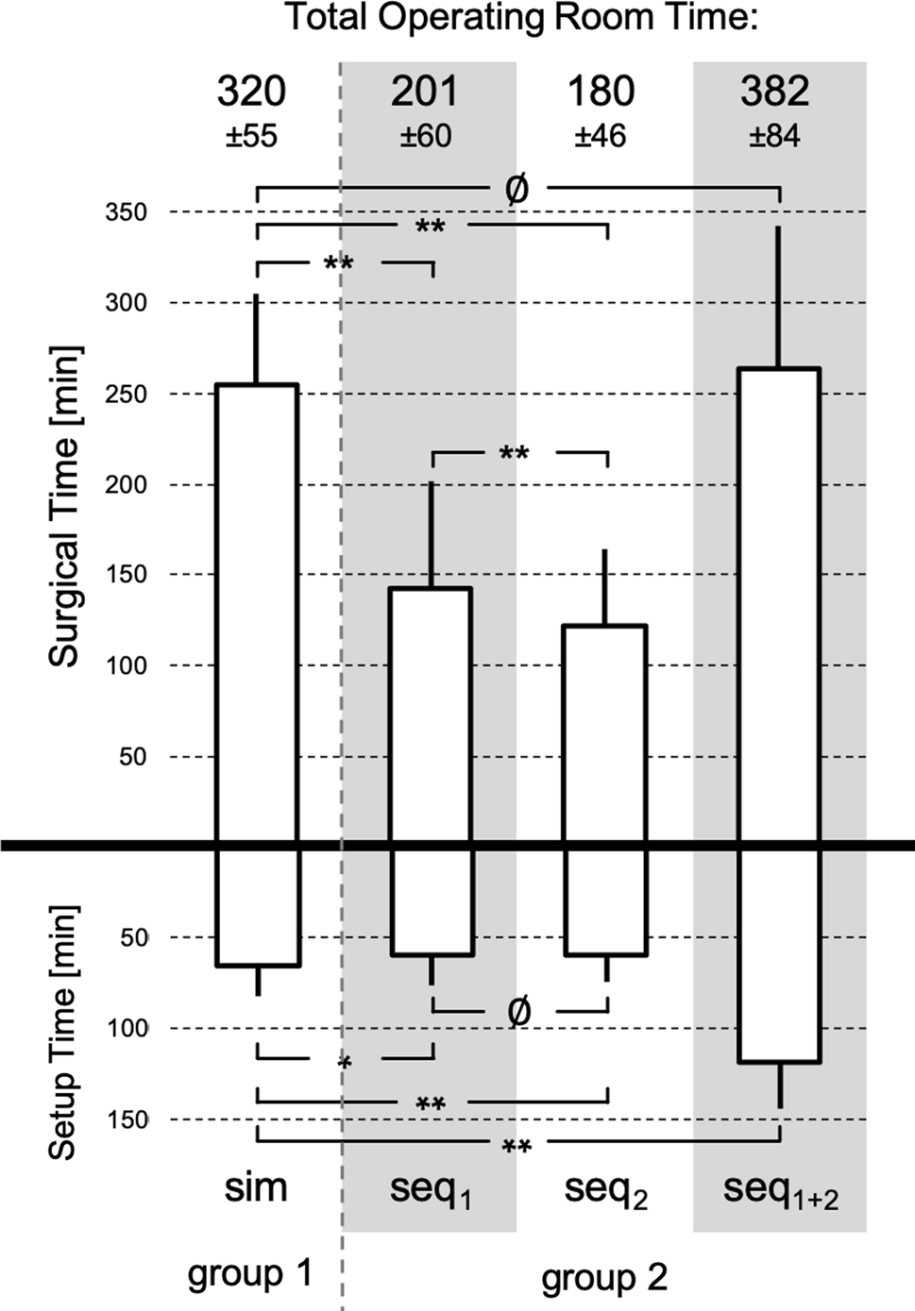
Duration of surgery in simultaneous (group 1) and sequential (group 2) bilateral cochlear implantation. The patients in group 2 are differentiated according to the two consecutive procedures on both sides (seq_1_ and seq_2_) and as an overall view (seq_1+2_). The mean value ± standard deviation of the surgical time, the setup time and the total operative room time (TORT) are shown. Statistically significant differences are marked with * (*p* < 0.05) and ** (*p* < 0.01) (ø = no difference)

The duration of the actual surgical activity (surgical time) was highly significant longer for simultaneous bilateral surgery in group 1 (255 ± 50 min) than for the individual sequential surgery. The surgical time of the second side in group 2 was highly significant shorter than that of the first ear (142 ± 60 min vs. 121 ± 42 min). There was no significant difference between the surgical time of the simultaneous surgery and the sum of both sequential surgeries (263 ± 79 min) (see Fig. 1).

Considering the unilateral first surgery (surgical time of group 2, first side), there was a weak negative correlation of the surgical time with the year of implantation (*r*(135) = − 0.264; *p* < 0.01). Thus, surgical time decreased by an average of 8.5 min per year. This effect was weaker for the surgery of the second ear. Further dependencies of the surgical time on implants form different manufacturers, surgeons or the age of patients could not be statistically proven.

The total operating room time in group 1 (320 ± 55 min) was highly significant above the TORT of the sequential surgery of group 2 (201 ± 60 min first ear and 180 ± 46 min second ear, respectively). In addition, the TORT in group 1 was highly significant below added TORT of the sequential surgery of both ears of group 2 (382 ± 84 min).

### Intra- and postoperative complications

#### Surgical complications

Complications in general occurred in 10/34 = 29.4% of patients from group 1 and 55/135 = 40.7% of patients in group 2. This difference was not statistically significant. 29/135 = 21.5% of patients from group 2 had complications after the first surgery, and 26/135 = 19.3% of patients were affected after the second implantation (*p* < 0.05). 4/135 = 3.0% of the sequentially implanted patients experienced complications during both implantations. 2/34 = 5.9% patients from group 1 had more than one complication postoperatively, respectively 4/135 = 3.0% patients in group 2.

**Fig. 2:**
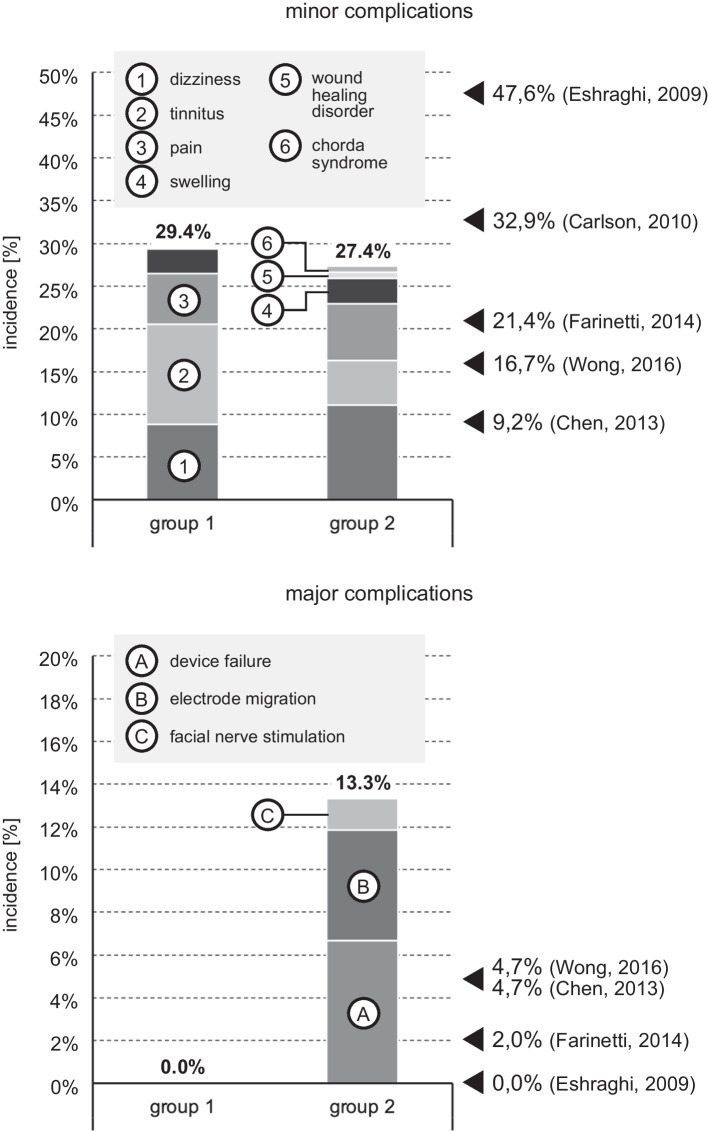
Incidences of minor (1–6) and major complications (**A**–**C**) in both groups. Arrow marks on the right margin indicate comparative values for the total complication rate from the literature. The differences between the two groups are not statistically significant

All complications in group 1 were minor complications with an incidence of 10/34 = 29.4%. The incidence in group 2 (37/135 = 27.4%) showed no significant difference. Dizziness was the most frequent complaint and occurred in 3/34 = 8.8% of the patients in group 1 and in 15/135 = 11.1% in group 2. In group 2 5/135 = 3.7% suffered from temporary dizziness after the first implantation and 10/135 = 7.4% after the second implantation. These differences were not significantly different. Most cases of dizziness were registered during the first two weeks after the surgery or immediately post-op (10 cases), the maximum time until onset of the symptom was 60.9 weeks (mean = 10.6 weeks). Other frequent complaints were tinnitus (4/34 = 11.8% in group 1 and 7/135 = 5.2% in group 2), pain (2/34 = 5.9% vs 9/135 = 6.7%) and swelling (1/34 = 2.9% vs 4/135 = 3.0%). In addition, in group 2 a mild wound healing disorder and a chorda syndrome occurred in 1 patient each (s. Fig. 2).

Major complications were significantly less frequent than minor complications. No major complications occurred in group 1. In group 2, major complications occurred in 18 patients (13.3%). In 9 cases, device failures were observed that led to explantation or reimplantation on the affected side. These events occurred at the earliest after 8 weeks and at the latest after 417.1 weeks (mean 133.6 weeks). In 7 of 9 cases, this involved the same batch of implants, which was particularly susceptible to technical failures. In 7 cases electrode migration occurred at a mean of 87.8 weeks after implantation which required revision surgery for reinsertion of the electrode and additional fixation of the electrode cable. 2 patients experienced co-stimulation of the facial nerve after 7.0 weeks and 46.1 weeks, respectively. In one case this led to explantation, in the second case the issue could be resolved by changing the processor settings.

**Fig. 3:**
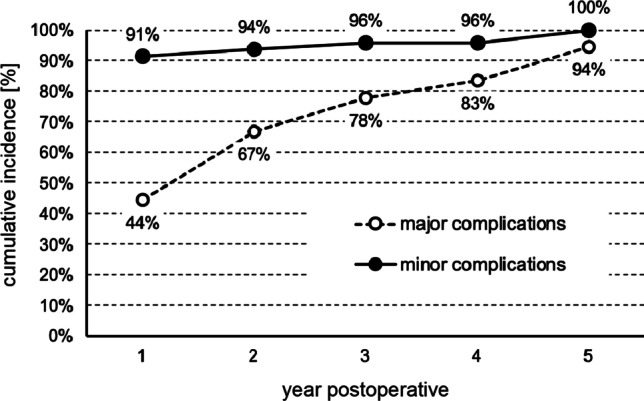
Cumulative incidence of minor and major complications over the course of the first 5 postoperative years of follow-up. 6% of major complications occured after the fifth year of follow-up

While most of the minor complications were registered during the first two weeks after surgery, the longest time span to onset was 248.6 weeks (mean = 20.7 weeks). Major complications occurred at the earliest 7 weeks after surgery, the latest recorded major complication was 417.1 weeks (mean = 103.9 weeks). The difference in mean time to occurrence between minor and major complications was statistically significant (*p* < 0.05). Figure 3 shows the time course of the cumulative incidence of minor and major complications.

#### Non-surgical complications

Intraoperatively, non-surgical complications did not occur in any case. During the postoperative phase however, in group 1, one case of fulminant pulmonary artery embolism with lethal outcome occurred: the patient collapsed during early mobilization less than 6 h after the end of surgery and required resuscitation. Despite immediate and maximal intensive medical treatment, the patient died the following day. A dependency of this tragic event on the group assignment could not be statistically proven despite the use of Fisher's exact test and even when taking all 270 individual interventions in group 2 into account.

Another patient from group 1 was diagnosed with nosocomial pneumonia on postoperative day 2, which healed without consequences after antibiotic therapy according to the guidelines.

One patient died of a severe complication of a pre-existing chronic disease (ARDS in the context of pneumonia under immunosuppression in exacerbated ulcerative colitis) 20 months after surgery, independent of his CI surgery.

### Duration of hospitalization

**Fig. 4:**
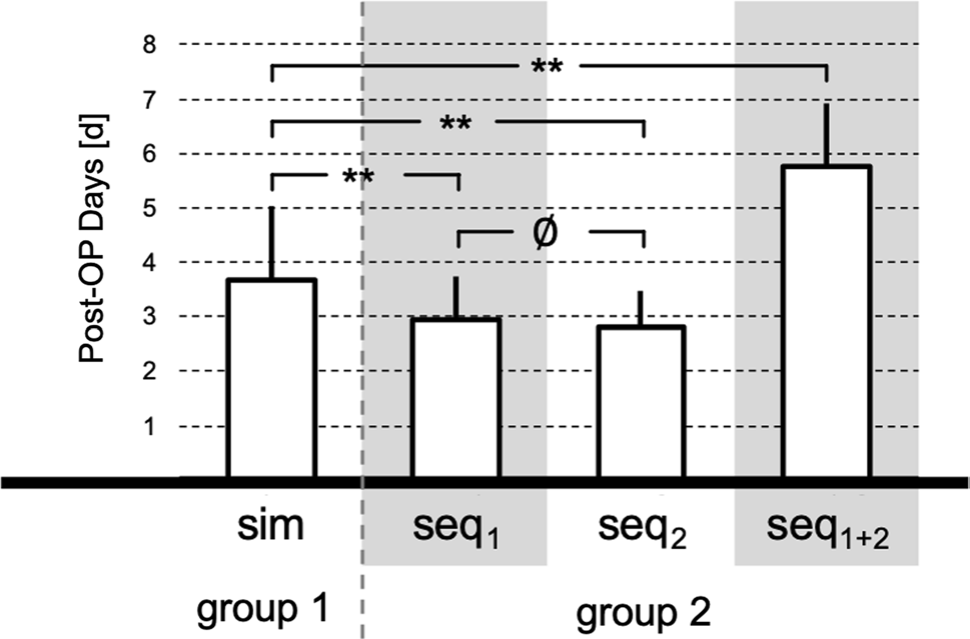
Duration of hospitalization. Mean ± standard deviation of the number of postoperative days of both groups. Statistically significant differences are marked with * (*p* < 0.05) and ** (*p* < 0.01) (ø = no difference)

Patients of group 1 spent at mean 3.7 ± 1.4 postoperative days in hospital. and were hospitalized on average 0.72 days longer than group 2 (*p* < 0.01). There was no statistically significant difference between the duration of hospitalization for the first and second surgery in group 2 (3.0 ± 0.8 days and 2.8 ± 0.7 days, respectively). In total, the sequentially bilateral implanted patients were hospitalized significantly longer than group 1 for the sum of both procedures (5.8 ± 1.2 days) (see Fig. 4).

Overstayers discharged after the third postoperative day were significantly more frequent in group 1 with 13/34 = 38% than in group 2 with 28/270 = 10% (*X*^2^ = 20.1; *p* < 0.01). Reasons for prolonged inpatient stay were partly transient minor complications (see above) and partly caused by comorbidities unrelated to CI surgery. These included chronic cardiovascular and pulmonary diseases and in one case a condition after severe traumatic brain injury. This patient suffered from bilateral acute deafness due to the trauma and required transfer to a neurological rehabilitation.

## Discussion

Patients with bilateral profound hearing loss or deafness who meet the audiological criteria for CI therapy benefit significantly from bilateral CI treatment. This offers advantages over unilateral surgery regarding speech understanding, both in quiet [25] and in noise [13], and reduction of tinnitus or hearing stress [12]. Nevertheless, in contrast to the care of congenitally deaf children, simultaneous bilateral cochlear implantation in adults is the exception rather than a standard procedure [26].

One possible reason for the reluctance to indicate simultaneous bilateral treatment may be safety concerns. For this reason, we retrospectively examined complications and complication-related factors in adult bilateral CI treatment with simultaneous or sequential CI surgery. Our results showed no significant differences between the two groups regarding age, sex ratio, and choice of CI manufacturer.

### Duration of surgery

The total operation time (TORT) consists of the setup time and the surgical time. The setup time of the simultaneous group 1 was significantly longer than the setup time of the first CI side of the sequential group 2 and even highly significant longer than the second implantation side. Possible reasons for this may be a more extensive anesthesiologic preparation of the patients in view of the longer expected duration of surgery or a prolonged recovery phase. However, the difference was on average only 6 and 7 min, respectively, and thus appears to be negligible. When considering the entire treatment process in the sequential group, the setup time adds up to an average of 119 min. This is not only highly significant, but also clearly longer than the setup time of 66 min, which is just half as long for the simultaneous procedure. Uecker et al. and Ramsden et al. found similar effects, namely a 10–40% longer setup time for simultaneous surgery, but a preponderance of total setup times for both sequential surgeries over simultaneous treatment [20, 23]. From a risk management point of view, it should be kept in mind that—irrespective of the anesthesia time—the potentially risky steps of induction of anesthesia, intubation and extubation are performed twice as often in the sequential group as in the simultaneous group.

The surgical time for simultaneous implantation, averaging 255 min, was 1.8 times longer than the first implantation in the sequential group and 2.1 times longer than the second implantation. In fact, implantation of the second ear took highly significantly less time than the first surgery: the difference amounted to a mean of 21 min. The cause of this phenomenon can only be speculated. In most patients in the sequential group, both implantations were performed by the same surgeon. It is possible that previous experience from the first procedure helped in performing the second procedure. However, we also observed a reduction in the duration of surgery on the first side over the study period (mean 8.5 min per year). This effect was highly significant. We suspect the cause to be the increasing standardization of the procedure and growing experience base not only of the surgeons but also of the nurses and the engineers performing the intraoperative measurements of the implant. This increasing professionalization could explain the shorter duration of the second implantation, which in some cases follows the first site in a long-time interval. If the surgical times of both sides in the sequential group are added, there is no significant difference to the simultaneous surgery, with a difference of just 8 min in the mean value and at the same time a high dispersion of the values. In contrast, other authors reported that the simultaneous surgery took significantly less time than the two sequential procedures [20, 23]. Apparently, multiple factors influence surgical time. Puram et al. found a prolonged surgical time when an otosurgeon in training ("trainee") was involved [27]. Our relatively long surgical time in the simultaneous group could have been caused by the renewed sterile draping in the head area before the start of the second side.

**Fig. 5:**
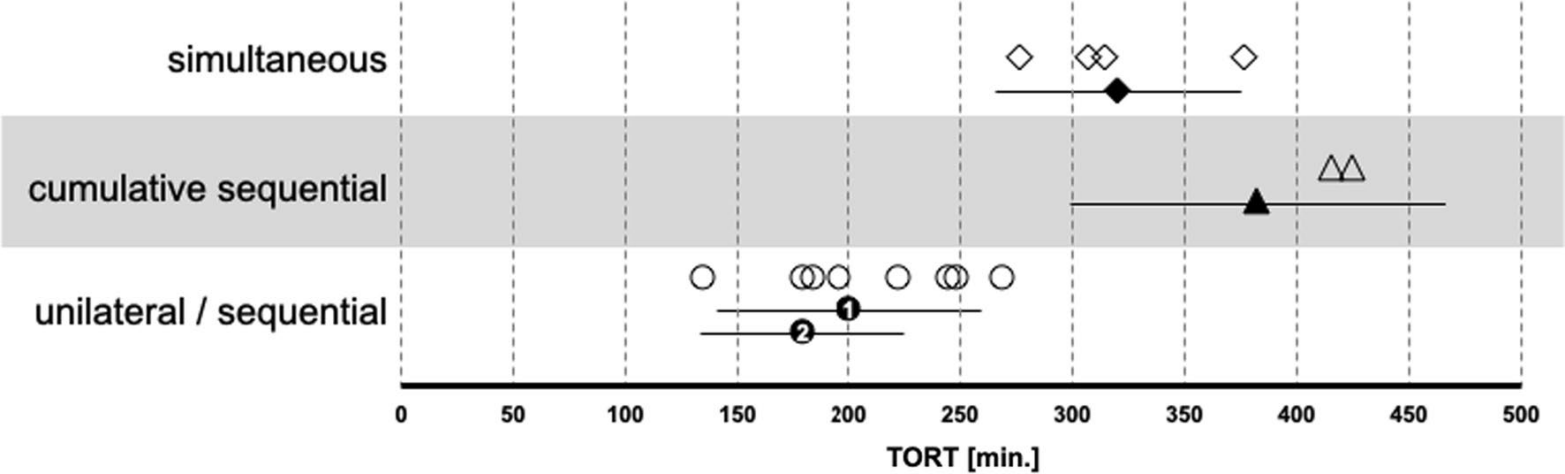
Comparison of the TORT of the present work (black marker: mean ± standard deviation) with the results of other authors (white markers: mean values) for the forms of care unilateral/sequential (circles, ⓵ = seq1, ⓶ = seq2), cumulative sequential bilateral (triangle) or simultaneous bilateral (diamond)

As a result of the shorter surgical time, the TORT (sum of setup time and surgical time) of the second implantation was also shorter than that of the first. Figure 5 compares the literature data on TORT with our own data. Despite the many factors influencing setup time and surgical time, the results for TORT are readily comparable: in the sum of both implantations in the sequential group, the surgery took about 60 min longer than with simultaneous approach. The reason for this is likely the double setup time. It remains speculative to what extent the double in- and extubation outweighs the possible risk of one long instead of two shorter surgical times. Our data show at least no inferiority of simultaneous CI treatment.

### Intra- and postoperative surgical complications

Complications in general (regardless whether minor or major) occurred with an incidence of about 30% in simultaneous group and 40% in sequential group (difference not statistically significant). These complication rates falsely suggest that cochlear implantation is risky, which is why a differentiated consideration is advisable: minor complications after cochlear implant surgery are quite common as they affected almost one third of the patients. The incidence was about the same in both groups with 29.4% for simultaneous and 27.4% for sequential bilateral treatment. Our data are in the middle of incidences reported in the literature [24, 28–30], which range from 9.2 to 47.6% (see Fig. 2). The obvious scattering of the incidences may be caused by heterogenous definition of certain complications or inconsistent precision in clinical documentation.

The most common minor complications in both of our groups were dizziness, tinnitus, pain and swelling occurring shortly and per definition temporarily after the procedure. Most patients complained about dizziness. One may hypothesize that simultaneous bilateral surgery affects both vestibular organs at the same time and therefore might come with a higher risk for dizziness. And indeed, the incidence in the simultaneous group was 8.8% but only 3.3% for the first implantation in the sequential group. However, surgery of the second side in the sequential group was associated with twice the incidence (7.4%), resulting in a total of 11.1% of patients experiencing temporary vertigo in the sequential group. This trend was not statistically significant. Thus, delayed sequential implantation is not gentler on the vestibular organ than simultaneous surgery. Accordingly, there were no statistically significant differences between the two groups for the other minor complications tinnitus, pain and swelling. Minor complications are closely related to the time of implantation: two thirds of minor complications occurred within the first two postoperative weeks. In contrast, only 8.5% became symptomatic after the first postoperative year.

Major surgical complications are distinguished from minor complications in most of the literature by the fact that the impairment is either permanent or requires surgical revision [31, 32]. Fortunately, no major surgical complication occurred in the simultaneous group. In the sequential group 18 individuals = 13.3% of the patients suffered from major complications. Across both groups, the incidence of major surgical complications was 10.7%, significantly higher than the 0% to 4.7% reported by other authors [24, 28–30]. Three aspects play a major role here:

- We observed 9 device failures. 7 of the defective implants belonged to the same batch of newly introduced implants. Without this implant batch, the failure rate would be at a level comparable to other literature data of 1.1% [24].
- Electrode migration is also an above-average complication with 7 cases = 5.2% in the sequential group or 4.1% in both groups. The phenomenon of electrode migration has been intensively studied at our institution in the past [33, 34]. Tests for electrode migration are routinely performed for years as part of CI follow-up [35]. Because of our focus on this complication and the targeted search for it, the incidence we recorded may have been higher than that reported by other authors.
- We attribute a significant influence on the relatively high incidence of major surgical complications to the study design chosen in the present study: major complications occurred on average after 104 weeks. The latest complication occurred at 417 weeks, i.e. 8 years after implantation. With a shorter follow-up period, we would have missed these events. According to our best possible search of the medical literature, a minimum follow-up period of 5 years is unique. Most authors allowed the follow-up phase to end after 4 years. In our population, we would have missed 17% of major surgical complications in this case. Of course, our approach is also insufficient per se, as even longer follow-up periods must inevitably add more adverse events. For practicality, we recommend a minimum follow-up period of at least 2 years to reliably detect two-thirds of major surgical complications.

Our data showed no statistically significant evidence that the complication rate is lower in simultaneous compared to sequential CI surgery. Thus, in adults both methods should be considered equally safe procedures.

### Non-surgical complications

Unfortunately, a 51-year-old female patient died on the day of surgery after simultaneous implantation. The cause was a fulminant pulmonary artery embolism during early postoperative mobilization. The lethal outcome could not be averted despite all immediate intensive medical measures. The case was retrospectively reviewed in detail: only grade I obesity (BMI 30) had been known as risk factor. Thrombosis prophylaxis had been performed according to the guidelines. Due to the short time interval between surgery and pulmonary embolism as well as the extent of the embolic event, an asymptomatic deep vein thrombosis already present preoperatively was also considered as a potential cause. As this was a single event, a statistical association with the simultaneous group could not be demonstrated. The question whether sequential implantation would have prevented this tragic complication is speculative and cannot be answered by our data.

### Duration of hospitalization

Patients with simultaneous bilateral cochlear implantation were hospitalized significantly longer than patients in the sequential group or their unilateral implantation. However, this difference was rather small, averaging 0.7 days. Local standard is discharge on postoperative day 3. Overstayers hospitalized longer than this period were significantly more likely to be in the simultaneous group than in the sequential group. The reasons for the prolonged stay were minor surgical complications (see above) and comorbidities. When both inpatient stays were considered, patients in the sequential group were hospitalized a total of 2.8 days longer. Hassan et al. stated, that extending the length of stay by one day increases the probability of catching a hospital acquired infection by 1.37% [36]. Thus, a reduction in the duration of hospitalization appears desirable.

### Strengths and weaknesses of the study

The present study is a retrospective analysis of the recorded treatment histories of our CI patients going back to the year 2008. The data quality of peri- and postoperative complications strongly depends on the documentation quality and may lead to an underestimation of the incidence of minor complications in case of insufficient documentation. Another factor is the ambiguous definitions of complications: while these are clearer for major complications (e.g., implant defect), "transient dizziness" or "transient postoperative pain" may be defined very different. This explains the wide scattered incidence of minor complications in the literature. The results of the present study are in the middle range and thus appear plausible and representative. The raw data required to reproduce the above findings cannot be shared at this time due to ethical reasons.

Compared to many other studies, our study design has a particularly long follow-up period of at least five years after surgical treatment. Thus, medium- and long-term complications are likely to be reliably assessed.

## Summary

In general, cochlear implantation is a low-complication therapy that can effectively and safely treat patients with profound hearing loss. It is undisputed that bilateral CI usage offers several advantages. In the synopsis of all considered complications and complication-relevant factors, it seems certain that there is no provable disadvantage in simultaneous bilateral implantation compared to the more established sequential care pathway in adults. TORT is significantly shorter, intubation and extubation are performed only once each, complication rates are comparable, and hospitalization durations are shorter. Because of the small number of subjects in the simultaneous group, we were unable to demonstrate statistically significant differences for complication frequency. Thus, at least an equivalence of both methods can be assumed. However, the potential side effects of the longer surgical procedure in simultaneous bilateral implantation have to be considered in each individual case. Careful patient selection with special consideration to existing comorbidities and preoperative anesthesiologic evaluation is essential. In summary, our data demonstrate that simultaneous bilateral CI surgery can be considered as a safe procedure for adults.
